# Pain in Mucopolysaccharidoses: Analysis of the Problem and Possible Treatments

**DOI:** 10.3390/ijms19103063

**Published:** 2018-10-08

**Authors:** Sabrina Congedi, Marcello Orzalesi, Chiara Di Pede, Franca Benini

**Affiliations:** 1 Dipartimento di Salute della Donna e del Bambino, Università degli studi di Padova, 35128 Padua, Italy; franca.benini@aopd.veneto.it; 2 Scuola di Formazione Maruzza in Cure Palliative Pediatriche “Maruzza Lyceum”, Fondazione Maruzza Lefebvre D’Ovidio Onlus, 00135 Rome, Italy; morzalesi@interfree.it; 3 Centro di Riferimento Veneto di Terapia del Dolore e Cure Palliative Pediatriche, Dipartimento di Salute della Donna e del Bambino, Università degli studi di Padova, 35128 Padua, Italy; chiaradipede@yahoo.it

**Keywords:** children, muchopolysaccaridoses, pain, treatments

## Abstract

Mucopolysaccharidosis (MPS) are a group of lysosomal storage disorders that are caused by the deficiency of enzymes involving in the catabolism of glycosaminoglycan (GAGs). GAGs incompletely degraded accumulate in many sites, damaging tissues and cells, leading to a variety of clinical manifestations. Many of these manifestations are painful, but few data are available in the literature concerning the prevalence, etiology, and pathogenesis of pain in children with MPS. This review, through the analysis of the data available the in literature, underscores the relevant prevalence of pain in MPSs’ children, provides the instruments to discern the etiopathogenesis of the disease and of pain, illustrates the available molecules for the management of pain and the possible advantages of non-pharmacological pain therapy in MPSs’ patients.

## 1. Introduction

Mucopolysaccharidoses (MPS) are a group of rare, inherited, lysosomal storage diseases that are caused by the lack, deficiency, or malfunctioning of the lysosomal enzymes involved in the degradation of complex carbohydrates known as mucopolysaccharides or glycosaminoglycans (GAGs) [1]. MPS disorders are autosomal or X-linked recessive inborn errors of metabolism and they are described as inherited neurometabolic disorders (iNMDs) [2]. Epidemiological studies report different data regarding MPS incidence: values of 2–3,5 per 10.0000 live births are mostly referred [3,4,5,6,7].

Patients with MPS are characterized by the accumulation of incompletely degraded GAGs in multiple sites that results in severe clinical manifestations and premature death. The storage products cause a cascade of events, leading to the progressive damage of cells, tissues, and organ systems. Most patients with MPS have a normal appearance at birth, but the progressive storage of GAGs in cells leads to a wide range of clinical manifestations. The lacking or non-functioning enzymes impact on the child’s growth and on the nervous system development from birth to adulthood, causing severe and irreversible neurological manifestations. Clinical features include facial dysmorphisms, dental abnormalities, hearing loss, ophthalmogical manifestations, joint and bone disease, cardiorespiratory problems, gastro-intestinal disorders, neurogical problems, and cognitive impairment [8]. The different types of MPS have been historically numbered from I to IX (Table 1): even if the clinical features are largely comparable, a wide range of phenotypes, different progression velocities, and grades of severity are reported for each type. 

In absence of specific treatments premature death occurs in most patients with MPS [9].

Cognitive delay is present in patients with MPS I type (or Hurler Syndrome), MPS III type (or Sanfilippo syndrome), and in the more severe form of MPS II (Hunter Syndrome) [1].

MPS is usually suspected clinically in early childhood and the diagnosis is confirmed by molecular biochemical analysis. The genetic analysis can identify a variety of different mutations for the genes involved, as well as carriers and new variants of unknown significance [10]. A timely diagnosis of MPS allows for planning an early global intervention and to prevent irreversible damages. From the time of diagnosis, a multidisciplinary approach should be undertaken, including clinical and psychological aspects and support to the patients and their families. Treatment options currently available are intended to guarantee a better quality of life, particularly when healing the disease is impossible. Specific enzyme replacement therapy (ERT) has been approved and it is currently available for MPS I, II, IVA, and VI, but only MPS IVa is treatable by ERT [11,12,13], and hematopoietic stem cell therapy (HSCT) is the main therapeutic option for MPS I Hurler patients under 2.5 years old [14]. 

In MPS patients, with and without cognitive impairment, pain is a common feature but it is still often unsuspected, poorly assessed, and undertreated. Few studies have fully assessed the prevalence of pain and its impact on the quality of life in this population. In the less recent literature, pain was considered only as a possible outcome measure of successful ERT) [15,16]. Brands et al. [11], examined pain prevalence in paediatric patients with all types of MPS. They conducted a survey in the Netherlands using questionnaires to evaluate different aspects of the disease: symptoms, developmental and disability level, and quality of life. In this study, pain was also evaluated in MPS patients with intellectual disability and a higher pain prevalence was reported (according to this survey, up to 53% of MPS patients with cognitive delay and only 30% of patients with a normal intelligence experienced pain) [11].

In this study we present a systematic review of the current knowledge regarding mucopolysaccharidosis in children, with the purpose to discuss etiopathogenesis and clinical features of the disease, paying particular attention to pain. In particular, we discuss the possible etiopathogenesis of pain and its pharmacological and non-pharmacological management.

## 2. Etiopathogenesis of the Disease

In order to explain and understand the etiology of MPS, studies on animal models have been conducted revealing massive storage of GAGs in liver, kidney, lung, spleen and brain [17,18,19]. Moreover, GAGs were also found in the trabecular matrix and bone cortex [20].

Many authors have investigated the specific pathways that are altered in MPSs: Lysosomal enzymes activity, gangliosides and cholesterol metabolism, and inflammation. In MPS I, MPS II, MPS III and MPS VII many authors have confirmed how the deficiency of a lysosomal hydrolase can lead to the imbalance of other lysosomal enzymes activity. This could be explained by the influence of a large mass of lysosomes in cells or by the stabilisation of enzymes by the storage material. Previous works revealed an increased activity of β-hexosaminidase, α-glucosidase, β-galactosidase, and β-gluronidase enzymes in tissues, such as liver and brain, while sialidase (also known as neuraminidase) and *N*-acetylglucosaminyltransferase were less active. In humans affected by MPS I, MPS II, and MPS IIIA, a significant alteration of ganglioside metabolism was also described: accumulation of glycosphingolipids, most notably GM2 and GM3 gangliosides, were reported [21,22,23]. This surprising feature was studied in animal models with MPS I, MPS II, MPS IIIA, MPS IIIB, MPS IIID, and MPS VI [18,20,23,24,25]. These studies suggest that glycosphingolipids storage is found in the early stage of the disease and it is one of the main elements that is involved in central nervous system (CNS) damage, causing alterations of dendritic cells, of axonal morphology, and of synapses. As a consequence, the ectopic dendritogenesis can lead to the formation of the so called “meganeurite” (a swollen section of the axon). These alterations are frequently observed in gangliosidosis, such as Tay-Sachs and Niemann-Pick [25,26,27].

Recent papers reported another feature in murine models of MPS I, MPS IIIA, and MPS IIIB: the accumulation of free unesterified cholesterol in neurons, secondary to ganglioside storage [26]. The altered intracellular distribution of cholesterol found in Niemann–Pick disease C [27] and glycosphingolipidoses GM2 [28] leads to a defective transport of glycosphingolipids; the accumulation of these molecules could explain the sequestration of membrane rafts and contribute to neurological damage [27,28]. Recently, inflammatory responses have been described as a prominent component of MPS disease. Inflammatory cascade in MPS I and MPS IIIB has been reported as a possible cause of CNS damage [29]; many elements are involved in this process (perineuronal microglial activation, increment of transcripts for CD38, lysozyme M, cathepsins S and Z, cytochrome b558, DAP12, and complements C1q and C4), all being related to an exaggerated macrophage/monocyte activation [30]. Evidence of the role of inflammation cascade has also been reported for bone and joint disease in MPSs. Musculoskeletal problems are prominent features in MPSs; even if no clinical signs of joint inflammation are present, previous studies conducted on animal models of MPSs I, II, VI, and VII reported local, intracellular inflammation due to dermatan sulfate storage.

Anatomopathological elements of cartilage apoptosis, synovial hyperplasia, recruitment of macrophages, altered connective tissue matrices, and inflammatory joint destruction are described in MPS patients as a consequence of dermatan sulfate accumulation. These damages are produced by dermatan sulfate storages through intracellular inflammation, alteration of growth of connective tissue and other cells, production and release of inflammatory cytokines, chemokines, proteases, and nitric oxide that induce the production of proapoptotic lipid ceramide [31,32]. Moreover, animal studies indicate that GAGs can inhibit collagenase activity of cathepsin K, with an alteration of chondrocyte maturation, mineralization, and osteoblast differentiation [33].

## 3. Etiopathogenesis of Pain

Pain is a common feature in MPS patients, but it is still inadequately assessed and undertreated. Few data are available on the physiopathology and epidemiology of pain in MPS children. We carried out a PubMed search of the literature while using the following mesh terms ““Pain/pathology” [Mesh] AND “Mucopolysaccharidoses”” and two items were obtained. When picking age filters and choosing the category “children: birth-18 years”, we did not find any publication.

In general, pain is typically classified into three major categories, applied for both adults and children: nociceptive (somatic or visceral), neuropathic and mixed [34].

Nociceptive pain (NOP) is due to the direct involvement of somatic superficial or deep tissue or visceral structures. This pain is referred as well localized with different characteristics due to its origin; it could be described as a fixed deep or as an acute stabbed pain. A review on lysosomal diseases [34] reported joint stiffness as a hallmark of almost all MPS, from mild-moderate forms to severe ones. This manifestation, which is not present in MPS IV and MPS IX [35], is probably due to GAGs storage in ligaments, tendons, joint capsules, and other soft tissues along with epiphyseal and metaphyseal deformities [7]. Joint swelling is frequently associated with joint stiffness and is due to bone enlargement [35]. This is an example of nociceptive somatic pain that is frequently reported in children with MPS; it could be caused by tissue injury or inflammation [36]. Nociceptive free-nerve endings and neuropeptides, such as substance-P, which is present in many articular and peri-articular tissues (accessory ligaments, synovium, subchondral bone, menisci, and periosteum) are the mediators that are involved in the origin of joint pain. Trigger finger, also known as stenosing tenosynovitis, caused by GAGs storage in the capsular tissues of joints or flexor tendons is frequently reported in MPS patients [37,38]. A role of the vascular system in pain generation, through vasospasm or ischemia, has been proposed but has yet to be proven [39]. Angina pectoris in children and infants has also been reported as a feature of MPS (Hurler’s syndrome) [40]. Indeed, in these patients a coronary arterial obstruction could be observed due to luminal narrowing and thickening of the arterial wall by partially degraded GAGs [41]. Another possible cause of visceral pain in MPS children is hepatomegaly or hepato-splenomegaly, being due to the abnormal accumulation of partially degraded GAGs [8,35,42].

Neuropathic pain (NP) is caused by injury, inflammation, or dysfunction of the peripheral or central nervous systems [43,44]; more specifically, it is secondary to the continuous stimulation of nociceptors as a result of chronic inflammation [45]. NP is referred as a stinging, stabbing pain, like a feeling of pins, associated to tingling and hyperalgesia, hyperaesthesia, and allodynia; it does not successfully respond to opioids, while anti-depressants (amitriptyline, nortriptyline) and anticonvulsant drugs (carbamazepine, gabapentin) are efficacious. Neuropathic pain is a common symptom associated with peripheral neuropathy and carpal tunnel syndrome can be a possible cause of neuropathic pain in MPS patients. Carpal tunnel syndrome is defined as an entrapment neuropathy of the median nerve at the wrist [46] and classic symptoms include numbness and paresthesias in the radial three-and-half-fingers of the hand, and motor weakness of the thenar muscles. These alterations in MPS patients are due to the compression of the median nerve due to thickening of the flexor retinaculum and the tissues around the nerve sheaths [47].

In patients that are affected by MPS, spinal cord injuries (SCI) are frequently reported: cervical myelopathy, paraplegia, and sudden death could be consequences of cervical stenosis and instability, due to hypoplasia, ligamentous laxity, incomplete ossification of vertebrae and extradural GAGs deposition [48]. Patients with SCI often develop chronic neuropathic pain [49]. Endothelin peptides, which exert their effects via endothelin A (ETAR) and endothelin B (ETBR) receptors, can contribute to this pain through an increased neuronal excitability in somatosensory pathways [50,51,52].

## 4. Epidemiology of Pain

Pain, in general, is a common symptom in children and adolescents, ranging from 20% to 46% individuals worldwide [53]. Pain is also common in MPS patients with and without cognitive impairment, but few data are available about its real frequency, probably because it is poorly assessed. The underestimation of pain in this population can be explained, at least in part, by a difficult clinical evaluation due to the complexity of the disease and the presence of cognitive impairment [54].

Musculo-skeletal problems are very common in MPS patients, mainly in type I, II, III, VI, and VII; joint alterations and skeletal deformities generally develop in the early-stages of the disorder [35,55,56]. In a survey that was conducted by Brands [11] and colleagues, the frequency and type of pain were investigated through questionnaires administered to children affected by different types of MPSs; in this group a pain score above the critical cut-off value for significant pain was reported in 40% of cases. The highest frequency of pain was observed in the MPS III group (52.9%); more than half of patients experienced joint pain (69%), mainly hip and back pain (27.8% and 25.9%, respectively). In a patient-reported outcome survey that was conducted by Hendriksz and colleagues [57], the impact of Morquio A syndrome (or MPSIVA) on adult and paediatric patients’ life was evaluated. The aims of the study were to evaluate quality of life, mobility, pain and fatigue. Joint pain was experienced by 64% of children and was inversely related to the intensity of wheelchair use. Those who frequently used a wheelchair reported less intense and less frequent pain than those using a wheelchair sporadically. The impact of wheelchair use on pain and fatigue followed a specific pattern: better mobility was associated with more pain and more fatigue. Hendriksz et al. identified in a better pain management and in the maintenance of functional capacity, elements that can significantly improve the quality of life in MPS patients [57].

Pain was also considered as an outcome measure in Morquio A patients by Burton et al. [58], who utilized the Word Graphic Rating Scale (WGRS), a visual analog scale (from 0 to 10): at baseline, a pain intensity score of 4.6 was reported. Musculoskeletal alterations are not usually considered principal features in MPS III patients, which are mainly characterized by neurodegenerative manifestations. However, although musculoskeletal manifestations are less severe in these patients, many families required an orthopaedic evaluation for the perceived musculoskeletal discomfort of hips and spine [59,60].

In the study published by White et al., several patients were referred to their service for hip pain, frequently associated with osteonecrosis of the femoral head (ONFH) [60]. De Ruijter et al. conducted a radiographic study in a group of MPS III patients, reporting a high prevalence of ONFH: Signs of ONFH were detected in eight patients (24%) and most of them (87%) referred hip pain [59]. Musculoskeletal problems, causing significant morbidity, were investigated by Vijay and Wraith [42] in a sample of 29 children with attenuated MPSI phenotype; in this survey, bone abnormalities were the prominent features at presentation and during the course of the disease (i.e., Hurler-Scheie or Scheie). Progressive arthropathy (86%), in particular located at the interphalangeal joints, with fixed flexion deformities of fingers (24%), was present; spine deformities such as gibbus (21%), kyphosis, scoliosis, and/or lordosis (24%) were also detected; carpal tunnel syndrome was present in more than half of patients (66%); recurrent ear, nose, throat (62%); and, airway inflammation (31%) were also found. All these manifestations are possible and concrete causes of pain; nevertheless, this symptom is not well investigated and extensive data are lacking.

Skeletal deformities, such as coxa valga and genua valga, are typical, frequent, and painful abnormalities in MPS patients, but no prevalence data are available [61]. Carpal tunnel syndrome (CTS) in children is strongly suggestive of MPS or mucolipidosis [62,63,64]. Hand pain, numbness, paresthesias, and a feeling of weakness in hand grip are the principal symptoms referred by children [65]. Cervical myelopathy and paraplegia must be always considered as possible painful conditions in MPS and they have been well documented in MPS IVA [66,67]. These conditions can be due to the undertreatment of upper cervical stenosis and instability and a prompt evaluation is needed in order to avoid severe nerve damage.

An interesting aspect is pain in MPS children with cognitive impairment; pain is more frequently reported in this group when compared to the group of children with normal intelligence. In the survey by Brands and colleagues, half of the mentally disabled patients presented pain compared to a third of children without a cognitive disability [11]. The high prevalence of pain in disabled children has been confirmed by other publications [68,69,70,71]. This high prevalence of pain in cognitively impaired patients could even be underestimated due to the difficulties of assessing pain in this particular population. The assessment and measurement of pain in children with cognitive delay must be performed utilizing pain scales validated for this specific population. Among the numerous tools available, the r-FLACC scale is preferred by parents and health workers [72,73].

## 5. Pharmacological Therapy

Children with MPS live with chronic pain and severe disability. Therefore, an adequate assessment and management of pain should be an essential component of paediatric care. MPS patients experience different types of pain (nociceptive, neuropathic, mixed) related to complications of the disease: skeletal deformities, muscular stiffness, compression of neural elements, and the compression of visceral organs. The main goals of paediatric pain management are the control pain and the improvement of the quality of life of children and their families.

Currently, two options are available: the treatment of the disease and the management of pain symptom. ERT and HSCT are the two available options that target the pathophysiological mechanism of MPS. The effect of HSCT on the patients’ reported health related quality of life has not been adequately addressed to date [74,75]. MPS patients (in particular, I, IVa, and VI) treated with ERT reported pain reduction and improvement in their quality of life and daily activities [13,16,76,77].

Laronidase (recombinant human alpha L iduronidase) is approved by the FDA as a specific therapy for MPSI. The approval of laronidasi is based on a phase III study on MPSI, in which patients were randomized to weekly intravenous infusions of laronidasi or placebo. After 26 weeks of treatment, patients in the laronidase group showed improved forced vital capacity and walking distance, and reduced hepatomegaly and GAGs as compared to the placebo group. In addition, laronidasi improved sleep apnea and shoulder mobility [78].

Gasulfase (recombinant human-acetylgalactosamine-4-sulftase) is approved by the FDA as a specific therapy for MPS VI [79]. This drug was administered once weekly and led to decreased urinary GAGs together with function improvement and prolonged survival [80]. In an open label phase II trial, patients who received weekly infusions for 48 weeks responded with improved endurance and decreased pain when compared with their baseline [14].

There is a critical need to understand the mechanism of chronic pain in MPS in order to improve its treatment. A potential target is inflammation. Animal studies suggest a possible role of tumor-necrosis factor-alpha (TNF-alpha) antagonists [31]. Polgreen et al. demonstrated that higher TNF-alpha levels are implicated in pain [81]. In their five-years prospective longitudinal study, 55 patients with MPSI, II and VI were enrolled and annually evaluated; 51 healthy controls were enrolled in a separated cross-sectional study of bone and energy metabolism. Pain and disability were evaluated through the Children’s Health Questionnaire-Parent Form 50 (CHQ-PF50). TNF-alpha levels were measured in 48 MPS (22 treated with HSCT, 24 with ERT) and in 51 controls. TNF-alpha levels were higher in MPS when compared with controls. Higher TNF-alpha levels were associated with increased pain and decreased physical function. This study suggests that anti TNF-alpha agents could be a useful adjunctive therapy of pain. Both non-opioid and opioid drugs are currently used in paediatric pain control (Table 2). The choice of analgesic is usually based on the type, source, severity, and duration of pain; personal experience can otherwise influence the choice.

Non-opioid analgesics include Acetaminophen and non-steroidal anti-inflammatory drugs (NSAIDs). These drugs are used in children with mild pain, but they can be used in association with opioids to treat moderate and severe pain. Acetaminophen is the most widely used antipyretic and analgesic drug for mild pain treatment, because it is generally free of adverse effects when administered in appropriate therapeutic doses. Nonsteroidal anti-inflammatory drugs (NSAIDs) include a group of molecules with different analgesic power. A powerful NSAID drug is Ketorolac, efficient in severe pain, such as renal colics and bone fractures; it should be used for short periods for the high risk of side effects. Ketorolac has a reversible inhibitory effect on platelet aggregation and it can cause dose-related gastric ulcerations, even when administered parenterally.

Less side effects were reported for NSAIDs with intermediate power, like Naproxene, Diclofenac, and Piroxicam: all of these drugs have similar safety profiles. They are frequently used to treat moderate pain, particularly osteoarticular pain, headache, and migraine attacks. Finally, Ibuprofen and Ketoprofen are NSAIDs with less power and should be prescribed for mild and moderate inflammatory pain.

Ibuprofen is the most utilized NSAID in children. Ibuprofen may be more effective than Acetaminophen in those situations in which inflammation is the major cause of pain. The use of Aspirin has been restricted because of its association with Reye’s syndrome.

Opioids are generally used in children with moderate and severe pain. Opioids have opium or morphine-like properties. They interact with one or more of the four identified opiate receptors of the central nervous system, resulting in a reduction of perceived pain. The selection of the specific opioid depends on the type, intensity, and duration of pain; in children with chronic moderate pain a weak oral opioid (e.g., Tramadol) or long-acting stronger opioids at low oral doses (e.g., Oxycodone or Methadone) can be used. Children with more severe pain need to be treated with strong opioids, such as Morphine, Oxycodone, or Fentanyl. Morphine is generally administered in children with episodic severe pain, while a sustained release preparation or agents with a long half-life (e.g., Oral Methadone) should be used if severe pain becomes chronic (e.g., Oxicontin or Transdermal Fentanyl).

A fearsome form of chronic pain is neuropathic pain, because it is often refractory to conventional pharmacotherapies. Adjuvant therapies, including antidepressants (Tricyclic) and anticonvulsants (Gabapentin and Carbamazepine), can be very helpful in treating neuropathic and mixed pain in children with MPS. This therapy has the goal to treat visceral hyperalgesia, central and peripheral neuropathic pain [82,83].

Glucocorticoids can be successfully utilized to treat pain deriving from hepatic distension; Bisphosphonates are used to treat pain from bone fractures.

Cannabinoids have been used for pain relief for centuries, although poor knowledge was available regarding their mechanism. In the last two decades, the efficacy of Cannabinoids in treating neuropathic pain has been tested in animal models and relevant information was obtained after the discovery of cannabinoid receptors and their endogenous ligands [84,85]. At present, the main evidence-based clinical use of Cannabinoids concerns adult patients with different types of pain, such as refractory chemotherapy-related nausea and neuropathy [86], neuropathic pain, spasticity in multiple sclerosis [87], and AIDS-related neuropathy [88]. Few good quality randomized trials indicate that Cannabinoids are an effective and safe treatment option for chronic non-cancer (predominantly neuropathic) pain [89,90].

Few papers reported positive and negative effects of Cannabinoids use for chronic pain in adults, and there are even less data for the paediatric population. Notwithstanding the benefits reported, adverse physiological and physical effects (psychomotor and cognitive impairment, anxiety and panic attacks, acute psychosis and paranoia, dry mouth, blurred vision, palpitations, tachycardia, and postural hypotension) may limit their therapeutic use in children [91,92].

## 6. Non-Phamarcological Therapies

Pain has negative effects on all aspects of health-related quality of life, including physical, emotional, social, and role functioning [93]; high levels of pain are often associated with anxiety and depression [94,95].

Even if there are significant gaps in the existing literature on non-pharmacological management of pain, positive results are reported. Non-pharmacological therapies should be taken into consideration in addition to the pharmacological ones mostly for illness associated emotional, social, and spiritual disturbances and in the case of chronic pain [96]. Although non-pharmacologic techniques can be proposed as stand-alone treatments, they are often used in a multimodal therapy background, with the aim to make pain control more effective [97].

Non-pharmacological treatments may be classified, as follows: (i) psychological interventions (distraction, stress management, hypnosis, and other cognitive-behavioural interventions); (ii) acupuncture and acupressure; (iii) transcutaneous electrical nerve stimulation (TENS); and, (iv) physical therapies (including massage, heat/cold, physiotherapy, osteopathy, and chiropractic).

Psychological therapies can be delivered individually or in groups to children and their families [98], and they are effective in reducing pain intensity and disability in children with chronic pain. The psychological approaches for pain management include cognitive-behavioural therapy, self-regulation strategies (hypnosis and biofeedback techniques, interventions aimed at producing behavioural changes, psychosocial support [29,30]), and educational programs.

Strategies, such as distraction, relaxation, breathing, visualization, imagery, modifying cognitive distortions, desensitisation, and changing negative thinking into positive ones should be thought to patients, to cope with their pain [99]. In order to successfully include psychosocial interventions in pain management, several barriers must be removed. First of all, patients and their parents should establish and maintain a good relationship with the health care providers. It is also necessary to be aware that cultural and financial factors play a principal role for the effectiveness of psychosocial interventions [100,101]. Cultural differences strongly influence the pain behaviour in individuals (it could be emotional, stoic, demanding, or vague) and also the frequency of administration of pain medications [102].

Acupuncture is the stimulation of specific points on the body and it is widely recognized as a therapeutic procedure to treat osteoarticular pain in adults [103,104].

The efficacy of acupuncture in the treatment of different conditions in children, such as migraines, abdominal pain, fibromyalgia and complex pain regional syndrome has been reported in two studies [105,106]. Electroacupuncture and acupressure are treatments based on acupoint stimulation: the first technique requires the delivery of electrical current through a needle; the second one requires acupoints stimulation through fingers and hands. Analgesia in acupuncture is realized through the modulation in central descending pain pathways (nucleus raphe magnus), influencing the release of endogenous peptides; the involvement of noradrenergic receptors has also been demonstrated in animal models [107,108].

In TENS, the nerves are stimulated by an electric current that is produced by a portable device inducing activation of non-nociceptive fibers thus obtaining pain relief (gate control therapy). Among physical therapies, many studies confirm the successful role of warming in treating acute and chronic pain: it can decrease the intensity of pain, anxiety, heart rate, and increase the pain threshold [109,110]. Positive results, such as reduction of anxiety and pain intensity, have been reported for massage and chiropratic interventions in children [111,112]. Few data are available regarding the effectiveness of music therapy in pain management: it has been used with positive results as part of multidisciplinary approach to treat chronic pain in children [113,114]. Among alternative therapies, an emerging strategy in pain control is the animal assisted therapy (AAT). A number of studies have suggested a positive effect of AAT in different health care settings but much less on pain in children. Braun et al. and Sobo et al. studied AAT and pain perception in children, finding a significant decrease in pain levels [115,116]. Further research is needed to prove the potential value of complementary therapies and their applicability in paediatric pain.

## 7. Surgical Treatment

During lifetime and from the early stages up to 75% of patients with MPS may need emergency or elective surgical interventions. However, surgery can only improve the signs of MPS disease expression due to GAGs’ tissue accumulation and not to effectively treat the disorder. Due to a variety of musculo-skeletal problems, MPS pediatric patients may benefit from a surgical approach to solve CPS, hip displasya [117], trigger fingers, odontoid dysplasia or hypoplasia, and severe scoliosis [118,119]. Furthermore HSTC does not arrest the progression of many disabling musculoskeletal abnormalities and orthopedic surgery should encourage to enhance quality of life but is also necessary to consider that anesthetic morbidity/mortality is not insignificant in these patients [119]. Facial deformities, megaloglossia, alteration of cervical and dorsal rachis, airway stenosis represent elements that strongly suggesting a difficult ventilation and/or intubation. Therefore, a preoperative assessment and simulation are needed and a multidisciplinary evaluation should be always performed to evaluate the effective advantages of elective surgical procedures in MPS patients.

### BOX 1

A 20-year-old girl with MPS IV is followed by our service for chronic cephalgia and low back pain, with a poor response to bethametasone and morphine. At inspection, we observed straight cervical rachis with a severe limitation of the range of motion (ROM); acupressure of dorsal and lumbar spinal apophysis worsened back pain. On the basis of the clinical history and physical examination, a mixed pain, both nociceptive and neuropathic, was diagnosed. Brain and rachis MRI were performed, showing anomalies due to neurological and skeletal involvement by cortical atrophy, cerebral gliosis, diameter reduction of spinal canal at C1–D7, diffuse herniated disks with spinal cord compression, and L5-S1 anterior spondylolisthesis. While considering the poor analgesic efficacy of morphine and its side effects, a cannabinoid therapy was successfully started with the association of corticosteroid and opioid treatment at need.

### BOX 2 

A 15-year-old boy with MPS-3A and severe cognitive impairment was referred to our Pediatric Pain Service for irritability of unknown origin. On examination tetraplegia with arms hypertonia and face grimaces, irregular breath, and moans after left leg mobilization were observed. These elements strongly suggested the presence of nociceptive joint pain. Pain intensity on the r-FLACC scale was 6/10. Acetaminophen was administered (15 mg/kg four times daily) with symptom resolution (0/10 r-FLACC scale) and a left leg subluxation was detected on X-rays. While considering the high anaesthetic risk, a conservative treatment with a timetable pain therapy was preferred.

## 8. Conclusions

MPS is a chronic disease that involves many organ systems. MPS children often experience chronic pain, but pain is often unsuspected, poorly assessed, and undertreated, and it has a deep negative impact on their quality of life. Effective therapeutic options are still limited and more specific, and targeted molecules are needed. Therefore, it is important to improve the understanding of the physiopathology of pain in order to improve its management and consequently the quality of life of MPS patients and their families.

## Figures and Tables

**Table 1:** Mucopolysaccharidoses (MPSs) from I to IX and their modality of inheritance, type of enzyme deficiency, and cosaminoglycans (GAGs) excreted and central nervous system (CNS) involvement.

MPS Disorder	Eponym	Inheritance	Enzyme Deficiency	GAGS Excreted	CNS Involvement
MPS I	Hurler/Scheie	AR	alpha-l-iduronidase	DS/HS	Hurler + Hurler-Scheie ± Scheie −
MPS II	Hunter	a X-linked recessive	iduronate-2-sulphatase	DS/HS	Severe form + Attenuated form −
MPS III A	Sanfilippo syndrome A	AR	Heparan *N*-sulfatase	HS	+
MPS III B	Sanfilippo syndrome B	AR	*N*-acetyl-a-d-glucosaminidase	HS	+
MPS III C	Sanfilippo syndrome C	AR	acetyl-CoA: a-glucosaminide *N*-acetyltransferase	HS	+
MPS III D	Sanfilippo syndrome D	AR	*N*-acetylglucosamine-6-sulfatase	HS	+
MPS IV A	Morquio A	AR	galactosamine-6-sulfatase deficiency	KS/CS	-
MPS IV B	Morquio B	AR	beta-galactosidase deficiency	KS	−
MPS VI	Maroteaux-Lamy	AR	arylsulfatase B	DS	−
MPS VII	Sly	AR	b-glucuronidase	DS/HS/C6S 1	+
MPS IX	Hyaluronidase deficiency	AR	hyaluronidase deficiency hyaluronidase none	None (2 Families described)	±

**Table 2:** Paracetamol, nonsteroidal anti-inflammatory drug (NSAIDs), and opioids dosage.

Molecule			Dosage
Paracetamol			*po*: 20 mg/kg initially, then 15 mg/kg every 4–6 h *rectal*: 30–40 mg/kg initially, then 15–20 mg/kg every 4–6 h *ev*: weight < 10 kg: 7.5 mg/kg every 6 h weight > 10 kg: 15 mg/kg every 6 h Maximum dose: 90 mg/kg/day (60 mg/kg/day if present risk factors)
Nsaid	Low Power	Ibuprofen	*po*: < 6 months: 5 mg/kg every 6–8 h > 6 months: 10 mg/kg every 6–8 h *rectal*: weight > 6 kg, 60 mg suppository every 8 h weight > 12 kg, 125 mg suppository every 8 h Maximum dose: 40 mg/kg/day
		Ketoprofen	*po, rectal or ev*: 3 mg/kg every 8–12 h Maximum dose: 9 mg/kg/day
	Moderate Power	Naproxene	*po*: 5–10 mg/kg every 8–12 h Maximum dose: 20 mg/kg/day
	High Power	Ketorolac	*po*: 0.2 mg/kg (max 10 mg) every 4–6 h *ev, im*: 0.5 mg/kg start, poi 0.2–0.3 mg/kg every 4–6 h Maximum dose: 3 mg/kg/day
		Indomethacin	*Po*, *ev*: 1 mg/kg every 8 hMaximum dose: 3 mg/kg/day
Opioids	Weak Opioids	Codeine	*po, rectal*: 0.5–1 mg/kg every 4–6–8 h *ATTENTION*: NO if < 12y–old NO for 12–18 y-old if: -recent tonsillectomy and/or adenoidectomy; -ultra-rapid metabolizer CYP2D6; -bad respiratory function
		Tramadol	po: 0.5–1 mg/kg every 4–6–8 h ev: 1 mg/kg every 3–4 h ev continuos infusion 0.3 mg/kg/h
	Strong Opioids	Morfina	CLORIDRATE *(ev*): bolus 0.05–0.1 mg/kg every 2–4 h, continuos infusion 0.02–0.03 mg/kg/h SOLFATE (*po*): early release: 0.15–0.3 mg/kg every 4 h; slow release: 0.3–0.6 mg/kg every 8–12 h
		Oxicodone	*po*: 0.1–0.2 mg/kg every 8–12 h
		Fentanyl	*ev*: bolus 1–2 gamma/kg (max 5 gamma/kg with spontaneous breathing), continuos indfusion 0.1 gamma/kg/h Intranasal: 1–2 gamma/kg
		Methadone	*po*: 0.05–0.1 mg/kg every 8–12 h (schedule modifying on the basis of duration therapy)

## Abbreviations

MPS	Mucopolysaccharidoses
iNMDs	Inherited neurometabolic disorders
GAGs	Glycosaminoglycans
ERT	Enzyme replacement therapy
HSCT	Hematopoietic stem cell therapy
ONFH	Osteonecrosis of the femoral head
NOP	Nociceptive pain
NR)	Neurophatic pain
CTS	Carpal tunnel syndrome
NSAID	Nonsteroidal anti-inflammatory drug
AAT	Animal assisted therapy
